# Probing the endosperm gene expression landscape in *Brassica napus*

**DOI:** 10.1186/1471-2164-10-256

**Published:** 2009-06-02

**Authors:** Yi Huang, Liang Chen, Liping Wang, Kannan Vijayan, Sieu Phan, Ziying Liu, Lianglu Wan, Andrew Ross, Daoquan Xiang, Raju Datla, Youlian Pan, Jitao Zou

**Affiliations:** 1 Plant Biotechnology Institute, National Research Council Canada, 110 Gymnasium Place, Saskatoon, SK, S7N 0W9, Canada; 2 School of Life Sciences, Xiamen University, 422 Siming Nan Road, Xiamen, Fujian, 361005, PR China; 3 Institute for Information Technology, National Research Council Canada, Ottawa, ON, K1A 0R6, Canada

## Abstract

**Background:**

In species with exalbuminous seeds, the endosperm is eventually consumed and its space occupied by the embryo during seed development. However, the main constituent of the early developing seed is the liquid endosperm, and a significant portion of the carbon resources for the ensuing stages of seed development arrive at the embryo through the endosperm. In contrast to the extensive study of species with persistent endosperm, little is known about the global gene expression pattern in the endosperm of exalbuminous seed species such as crucifer oilseeds.

**Results:**

We took a multiparallel approach that combines ESTs, protein profiling and microarray analyses to look into the gene expression landscape in the endosperm of the oilseed crop *Brassica napus*. An EST collection of over 30,000 entries allowed us to detect close to 10,000 unisequences expressed in the endosperm. A protein profile analysis of more than 800 proteins corroborated several signature pathways uncovered by abundant ESTs. Using microarray analyses, we identified genes that are differentially or highly expressed across all developmental stages. These complementary analyses provided insight on several prominent metabolic pathways in the endosperm. We also discovered that a transcription factor *LEAFY COTYLEDON *(*LEC1*) was highly expressed in the endosperm and that the regulatory cascade downstream of *LEC1 *operates in the endosperm.

**Conclusion:**

The endosperm EST collection and the microarray dataset provide a basic genomic resource for dissecting metabolic and developmental events important for oilseed improvement. Our findings on the featured metabolic processes and the *LEC1 *regulatory cascade offer new angles for investigation on the integration of endosperm gene expression with embryo development and storage product deposition in seed development.

## Background

Fertilization in angiosperms is a tale of two unions. In addition to the fertilization of the egg, which initiates the genesis of the diploid embryo, a second sperm unites with the two nuclei of the central cell to form the endosperm. The triploid nature of the endosperm renders itself a genetic dead end, but its nurturing character is evident both in anatomy and in metabolism. In most angiosperm species, early phases of embryo development proceed within an often partially "liquid" endosperm. Biochemical studies have demonstrated a role of endosperm in nutrient delivery from the parental plants to the embryo [1]. Not surprisingly, variations in the genetic constituent of the endosperm greatly impact the phenotype of seeds [2,3]. There has also been emerging evidence of cross talk between the maternal testa and the zygotic endosperm to influence seed size [4-6]. But until recently, attention on endosperm gene expression profiles has largely been focused on species in which the endosperm accumulates storage reserves. Transcript profiling studies in the endosperm of corn [7], wheat [8] and castor bean [9] have been reported. An array constructed from developing *Arabidopsis *seeds enriched for proliferating endosperm was successfully used to identify endosperm-expressed and endosperm-preferred genes [10]. But the development and metabolism of endosperm in exalbuminous seeds in general have not been studied intensively with modern genomics techniques.

*Brassica *crops are among the oldest cultivated plants, with written records dating back to as early as 1500 BC [11]. Cultivated for its premium oil, *Brassica *currently comprises the world's third largest oil-producing crop after soybean and oil palm. Because *Brassica *are close relatives of *Arabidopsis*, studies targeting *Brassica *plants directly benefit from the enormous knowledge base that is currently available in the public domain. Since storage product biosynthesis takes place predominantly in the expanding cotyledon, the embryo has been a focal point of genetic and metabolic studies of seed development. Consequently, several prominent regulators influencing cotyledon development and metabolism have been identified, including LEC1, which is involved in regulating cotyledon identity, and WRINKLED1 (WRI1) that regulates primary metabolism concerning storage lipid deposition.

In mature *B. napus *seeds, the endosperm is depleted to a single aleuronic layer in the mature seeds. This illustrates the dramatic cellular and biochemical shifts the endosperm undergoes during seed development. An improved understanding of the genomic landscape operating in the endosperm will facilitate both seed biology study and crop improvement efforts. In this study, we report results from an integrated analysis of developing *B. napus *endosperm based on ESTs, protein profiling and microarray datasets. We consider these efforts a first step to understanding the genomic landscape of developing endosperm and uncovering gene expression and metabolic events associated with seed traits of this important oilseed crop.

## Results and discussion

### Endosperm tissue collection and study design

**Figure 1:**
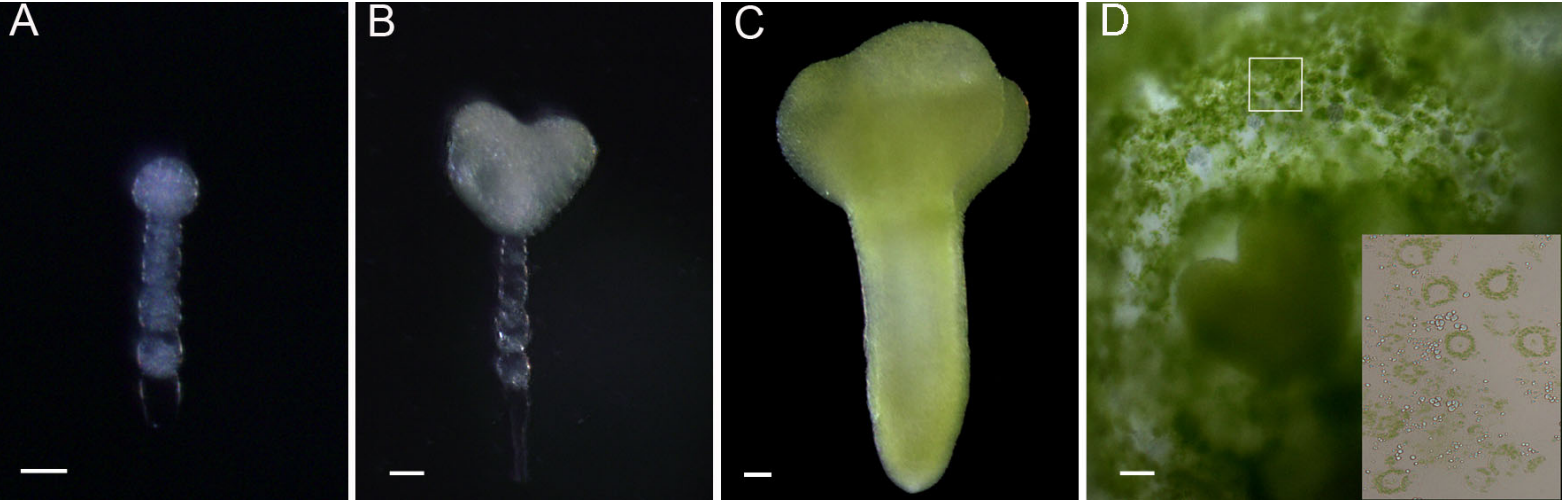
**Representative embryo development stages of *B. napus***. (A) globular-shape embryo stage; (B) heart-shape embryo stage; (C) cotyledon stage; (D) sectioning of a seed with heart-shape embryo is shown to illustrate the green endosperm tissue. Abundant chloroplasts surrounding central vacuole of the harvested endosperm cells are clearly visible (insert). Bars = 50 μm.

Similar to *Arabidopsis *[12], the development of seeds within a single silique in *B. napus *varies significantly in terms of developmental stages. Therefore, in this study we defined the developmental phase of the endosperm based on the stages of the embedded embryos. Under our growth conditions, the globular-shape embryo stage is reached approximately six days after flowering (DAF), the heart-shape embryo stage is reached after eight days, and by day 14 the seed enters the cotyledon stage (Figure 1). We collected endosperm tissues by first dissecting out the embryos, which were examined to determine the developmental stage. Then, using a glass pipette with a fine tip, the endosperm was extracted and collected into pools based on whether embryo development was at the globular-shape, heart-shape or cotyledon stages (Figure 1). During this process, care was taken to avoid tissue from the integument, but limited contamination cannot be ruled out. Amongst different sections of the endosperm, our tissue collections were enriched with micropylar endosperm surrounding the zygote (free nuclear endosperm). The endosperm tissue was photosynthetically active as clearly evident by the green endosperm cells shown in Figure 1D. Furthermore, it could also be seen that the harvested endosperm cells were laden with chloroplasts around a central vacuole (Figure 1D, insert). The collected tissues allowed the construction of two cDNA libraries: one derived from endosperm of heart-shape embryo stage seeds and the other from endosperm of a mix of globular-shape embryo and cotyledon stage developing seeds. Because the number of ESTs was not sufficient to justify separation into different pools, we combined the data into one endosperm database. For protein profile analysis, we focused only on endosperm in ovules containing heart-shape embryos because they exhibit all phases of endosperm development [13]. For microarray analysis, endosperms were harvested from seeds at the globular-shape embryo, heart-shape embryo and cotyledon stages, respectively.

### Endosperm EST Database

#### General description of the endosperm EST dataset

We sequenced 30,239 ESTs and a total of 24,881 high quality sequences were recovered. The entire EST data was deposited in dbEST of NCBI under the accession numbers from ES986411 to ES999999 and EV000001 to EV009938. Clustering with an overlapping length of 49 bases, a minimum effective length of 50 bases and similarity at 94% yielded 2,894 tentative contigs and 7,069 singletons, which represent a total of 9,963 unisequences. The average length of the unisequences was 501 bases (Table 1).

**Table 1:** Summary of the EST analysis.

Description	Number
Total ESTs	30,239
Total high-quality ESTs	24,881
Success index (%)	82.3
Number of contigs	2,894
Number of singletons	7,069
Number of unigenes	9,963
Average insert size (bp)	501
Average of GC percentage (%)	44.8

All unisequences were subjected to search by BLASTX against the *Arabidopsis *protein database at The *Arabidopsis *Information Resource (TAIR) for transcript homologs at an *E *value of less than 1e^-5 ^[14], and the accepted annotations had at least one high scoring pair (HSP) of minimum length 30 covering at least 75% of the query sequence with 80% identity. Approximately 72.8% (5,149 out of 7,069) of the singletons and 84.2% (2,436 of 2,894) of the contigs had *Arabidopsis *homologs. Among these unisequences, 9.6% (952) showed similarity to proteins of hypothetical or unknown function, 19.3% (1,920 of 9,963) singletons and 4.6% (458 of 9,963) contigs had no significant similarity to any protein entries in the database. Overall, 33.4% (3,330 of 9,963) unisequences expressed in *Brassica *endosperm were similar to proteins of unknown function. When the unisequences with *Arabidopsis *homologs were subjected to functional category classification through the Gene Ontology (GO) classification scheme [15], 90% were classified with GO annotation as involved in biological process, 92% as having molecular function and 85% as functioning as cellular components. The list of each unisequence identified and its best BLASTX match annotation is provided in Additional file 1. Records of analysis for these collections can be accessed at the FIESTA database developed at the Plant Biotechnology Institute, National Research Council Canada [16].

A report on an *Arabidopsis *EST dataset from whole seeds indicated that storage proteins represent over 50% of cDNAs in seed EST libraries [17]. As a quality control measure for tissue sampling, we searched ESTs for storage protein genes. We found only two unisequences corresponding to storage protein genes, representing less than 0.01% of our endosperm EST libraries. This indicates that contamination from embryo tissues in our tissue sampling is negligible.

#### Contigs of highly abundant ESTs

Because we constructed a non-normalized cDNA library, the abundance of ESTs from a given contig provides a relative measure of the expression level of the gene in the endosperm, albeit not in a stage-specific manner. We defined contigs derived from 10 or more ESTs as highly abundant (see Additional file 2).

The most abundant ESTs were from genes homologous to the *Arabidopsis *plastocyanin-like domain-containing protein genes, including AT2G23990, AT3G09390, AT4G31840 and AT4G32490. These genes, which are predicted to encode cell surface glycosylphosphatidylinositol (GPI)-anchored proteins [18], are represented by a total of 241 ESTs in our database. The encoded gene products have secretion signals and are thought to be extracellular proteins that are attached to the plasma membrane through the GPI anchor and function as mobile electron carriers. The second most abundant group of contigs encodes putative seed-specific lipid transfer proteins (LTPs) capable of binding lipid molecules under certain *in vitro *assay conditions [19]. A third group of rich ESTs is predicted to encode a membrane galactosyltransferase (AT4G32110), possibly involved in protein amino acid glycosylation or in reversible galactosyl transfer from galactinol to D-ononitol (lo-4-*O*-methyl-rnyo-inositol). Based on information available from the integrated *Arabidopsis *e-FP browser [20], the *Arabidopsis *homologs corresponding to these EST groups are all highly expressed during seed development. Two genes previously identified as highly expressed in castor bean endosperm [9], encoding a Metallothionein 2A homologous to AT3G09390 and a translationally controlled tumor protein homologous to AT3G16640, were also among the most highly abundant in our endosperm EST library.

Reflecting a generally active metabolic status, ESTs for genes encoding major biochemical components for redox control, including glutathione S-transferase (GST6), glutathione dehydrogenase/dehydroascorbate reductase (DHAR1) and peroxidase (PRXR1), were highly represented in our EST collection. Consistent with abundant chloroplasts observed during our tissue collection, ESTs related to photosynthetic activities were also among the most frequently detected groups. When compared with the list of the most abundant contigs in the *Arabidopsis *whole seed EST dataset [17], we found that nucleoside diphosphate kinase 1 (NDPK1), and myo-inositol-1-phosphate synthase (MI-1-P SYNTHASE) were conspicuously present in the endosperm-enriched EST collection, but not in the whole seed EST sets. Also noteworthy was the pronounced presence of ESTs for protein ubiquitination, including ubiquitin-protein ligases AHUS5 (also known as EMBRYO DEFFECTIVE 1637) and UBC2, ubiquitin 14 (UBQ14) and ubiquitin 1 (UBQ1, AT3G52590), to which the phenotype of EMBRYO DEFECTIVE 2167 was attributed. Additionally, genes encoding ADP-ribosylation factor (ARF) family proteins were also highly expressed. These genes participate in multiple intracellular trafficking events and have a central role in vesicle formation during early and late secretory trafficking [21]. Two vacuolar calcium-binding protein encoding genes (AT1G12080 and AT1G62480), likely reflecting a key role of the endosperm in mineral storage and transfer, were also among the most abundant ESTs (see Additional file 2).

We were surprised to find that two genes involved in transcription activations, *LEC1 *and *ANGUSTIFOLIA3 *(*AN3*)[22], were among the most highly expressed in the endosperm. In fact, ESTs derived from *LEC1 *was one of the top 20 ESTs in the endosperm. This will be further addressed below.

### Protein Profile Analysis

We focused on endosperm tissues harvested from seeds with heart-shape embryos for protein profile analysis. By combining one-dimensional gel electrophoresis with matrix-assisted laser desorption/ionization-time of flight mass spectrometry (MALDI-TOF MS) and peptide mass fingerprinting (see Methods), a total of 809 endosperm proteins were identified. The protein sequence data reported in this paper appears in the PRIDE database under the accession number 9198 [23]. A list of proteins whose functional descriptions were predicted based on their homologs from *Arabidopsis *is provided in Additional file 3. Protein entries were tabulated in order of high to low abundance because proteins with smaller hit numbers and larger protein scores/matches generally have higher abundance. Among the identified proteins, 28 were products of genes encoded in the chloroplast and mitochondrial genomes.

The protein profile data recapitulates some of the findings from the EST datasets, as illustrated by the numbers of ESTs corresponding to each of the protein entries listed in Additional file 3. The corresponding genes of about half (52%) of the identified proteins were represented by contigs with three or more ESTs. There was a sizeable presence of housekeeping components such as ribosomal proteins, proteins involved in cytoskeleton organization, molecular chaperones, and translation initiation and elongation factors. The protein profiles are notably enriched for enzymes involved in carbon fixation and glycosis/gluconeogenesis. Major starch and sugar metabolism enzymes, including sucrose synthase, starch synthase, hexokinase, triosephosphate isomerase, and several subunits of the ADP-glucose pyrophosphorylase, were all detected. Other prominent biochemical pathways whose enzymes were highly represented in the profiles include the tricarboxylic acid (TCA) cycle, redox homeostasis, and nucleotide sugar metabolism. We observed several enzymes involved in inositol phosphate biosynthesis, the metabolic branch concerning methionine synthesis and one carbon cycle metabolism. These results are all consistent with the EST dataset.

Concerning cellular events, there was an apparent enrichment for proteasome components, as well as biochemical machinery for protein ubiquitination, suggesting a high demand for protein turnover during endosperm development. In addition, we found many coatomer proteins, which are the principle components of the coat protein complex involved in endoplasmic reticulum-to-Golgi vesicle-mediated transport. A putative cell division cycle protein 48 (CDC48; AT5G03340) involved in membrane trafficking [24] was also found. It was also significant that our protein profile detected the homolog of lysophosphatidylcholine acyltransferase, the expression of which was revealed in our EST analysis. The fact that we detected the expression of this gene in both approaches suggests that it may play a significant role in endosperm development, likely in membrane lipid turnover [25].

It is interesting to note that we saw multiple isoforms of subtilase (subtilisin-like protease) family proteins [26]. It has been reported in *Arabidopsis *that some subtilisin-like protease genes are strictly expressed in the micropylar endosperm surrounding the embryo and are important for the cuticle development of the embryo [27]. We were also interested in looking for metabolite transporters that are potentially involved in nutrient trafficking. Only one integral membrane protein similar to an *Arabidopsis *sugar transporter ERD6-like 4 (AT1G19450) was found. This putative sugar transporter is located in the vacuolar membrane and is possibly involved in transporting sugars out of the vacuole under certain conditions.

### Microarray Analysis

#### General description of microarray data

**Figure 2:**
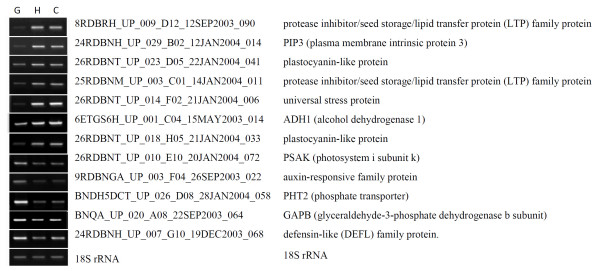
**Patterns of 12 unisequences that were identified by microarray and validated with RT-PCR**. (G) globular-shape embryo stage; (H) heart-shape embryo stage; (C) Cotyledon stage.

**Figure 3:**
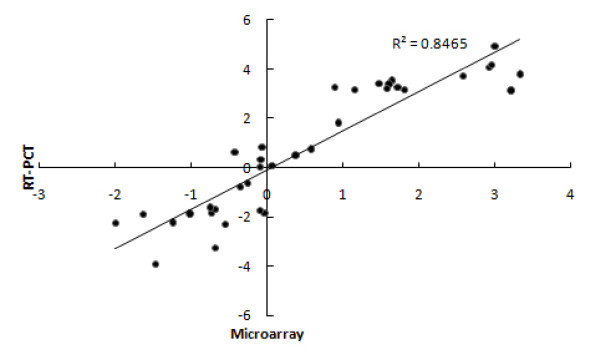
**Correlation of gene expression ratios between cDNA microarray and RT-PCR**. The gene expression ratios between tissue-pair comparisons were transformed with log2 ratio.

An in-house cDNA array system was used in this study (see Methods). The system consists of 10,642 amplicons of unisequences derived from cDNA libraries from several developmental stages of *Brassica*. This platform was deposited in Gene Expression Omnibus (GEO) database, under accession number GPL8090. The quality of the array was previously evaluated with different RNA sources [28]. Approximately 55% of the unisequences in our endosperm EST dataset were covered in this cDNA array. Two-color labeled cDNAs from endosperm at three embryo developing stages including globular-shape embryo, heart-shape embryo and cotyledon were used for pairwise comparisons. Two biological repeats and four technical repeats with color-swap hybridizations in each comparison were performed. Details could be accessed in Gene Expression Omnibus with accession number GSE14766 [29]. The reproducibility of the microarray hybridization and data quality were assessed through correlation analyses and RT-PCR. The correlation coefficient between duplicate spots within an array ranged from 0.97 to 0.99 and the correlation coefficient of the signal ratio for each tissue pair between technical repeats with same color labeling varied from 0.94 to 0.99. Furthermore, 12 unisequences were selected and subjected to RT-PCR. The expression levels of these genes covered a wide spectrum, thereby allowing us to compare the resolution and relative accuracy of microarray data. There is good correspondence (r^2 ^= 0.846) between the RT-PCR log2 ratio and the microarray log2 ratio (Figure 2 and Figure 3). These results confirmed the overall reliability of the microarray expression data.

**Figure 4:**
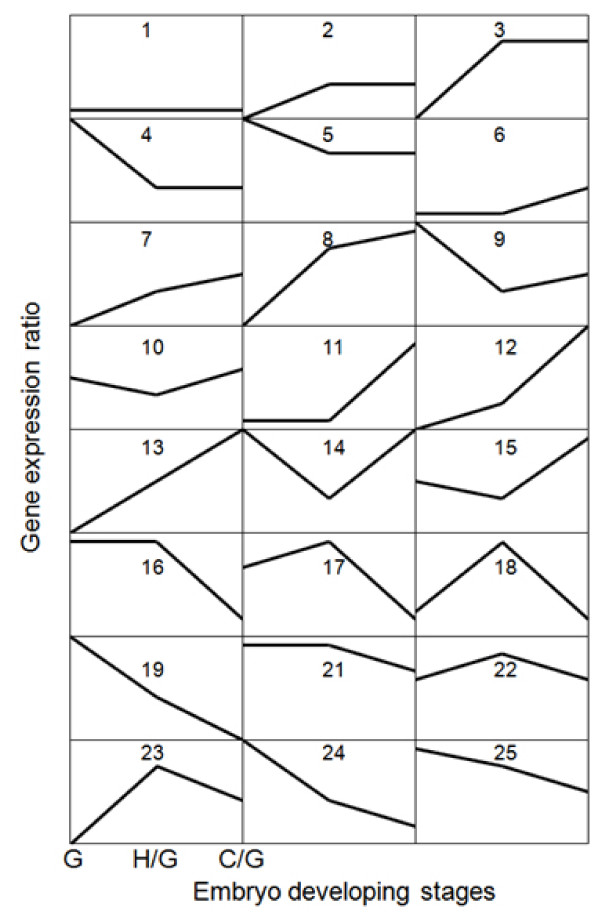
**Schematic description of the 24 patterns based cluster analysis**. The 1229 differentially expressed unisequences were classified into 24 clusters using a hierarchical algorithm. The mean signal ratio (in log2 scale) for unisequences in each cluster is plotted on the *y *axis. The globular-shape embryo stage was used as the reference point; the ratios of the heart-shape embryo vs. globular-shape embryo and cotyledon vs. globular-shape embryo are on the *x *axis from left to right, respectively. The number in each panel indicates the cluster ID.

The microarray data were normalized and went through rigorous preprocessing and filtering (see Methods). Using Rank Product [30] we identified 1,229 unisequences that were significantly differentially expressed during endosperm development (see Additional file 4). Twenty-four clusters were identified using a pattern-based clustering technique [31] (see Additional file 5). Clusters 3 and 4 (Figure 4) are two major clusters that collectively include 63% of all differentially expressed unisequences. Their patterns of change are mirror images of each another. The distribution of GO annotation in biological process for each cluster was calculated (see Additional file 6).

#### Identification of developmental stage favored genes

Certain embryo stage favored groups can be identified from the clusters as detailed in Additional file 7. The 24 clusters can be grouped into four classes: globular-shape embryo stage favored, heart-shape embryo stage favored, cotyledon stage favored, and others that do not fall into any of the above groups (Figure 4). In general, differential expression was most evident at early stages of endosperm development between the globular-shape and heart-shape embryo and the globular-shape embryo and cotyledon stages. The difference in gene expression between heart-shape embryo and cotyledon stages was not as obvious.

Genes of the globular-shape embryo stage favored class, which include clusters 4, 5, 9, 19, 24 and 25, show peak expression at the globular-shape embryo stage. Most genes involved in photosynthesis, such as light-harvesting complex proteins and chlorophyll-binding proteins, were among this group. A significant number of genes (55%) in this class still have unknown functions.

Clusters 17, 18, 22 and 23 constitute the heart-shape embryo stage favored class. Genes in these clusters showed an increase in transcript level from globular-shape embryo to heart-shape embryo stages, whereas the transcript level in endosperm at the cotyledon stage was generally lower than that at the heart-shape embryo stage. This group includes genes for biochemical machineries of protein turnover, such as ubiquitin-conjugating enzyme 16 (UBC16), ubiquitin 11 (UBQ11), cysteine proteinase, and heat shock proteins such as HSP70B, HSP101, and DNAJ. Genes encoding pectinesterase family proteins (CN732993 [*Brassica *EST accession], AT5G47500 [its Arabidopsis homolog], hereinafter denoted same) and cellulose synthase (CN727050, AT5G09870) also reached high expression levels in endosperm at the heart-shape embryo and cotyledon stages. Similarly, four genes encoding cellulose synthase (CESA1/CESA2) and α-1,4-glucan-protein synthase (RGP4) were up-regulated at this particular stage. We also identified two genes encoding auxin-responsive proteins and one for gibberellin 20-oxidase, whose expression levels increased from the heart-shape embryo to the cotyledon stages. The up-regulation of these genes suggests that these plant growth regulators may play a key role in regulating endosperm development at the heart-shape embryo stage.

The cotyledon stage favored class encompasses clusters 7, 8, 11, 12 and 13, which reached their peak expression levels at the cotyledon stage. Most prominent in this group are transcription factors, some of which are known to play a role in cotyledon development and storage product synthesis. These include *LEC1 *(CN732092, AT1G21970), *basic Leucine Zipper 25 *(*bZIP25*, EE436021, AT3G54620), *HIGH MOBILITY GROUP AT-hook *(*HMGA*, ES265203, AT1G14900) and *WRI1 *(DY013242, AT3G54320), which were up-regulated by 2.5- to 9.4-fold. Genes for myo-inositol-1-phosphate synthase (MI-1-P synthase, CN737485, AT4G39800) and the starch degradation enzymes, α-amylase (AMY1, CN727248, AT4G25000) and β-amylase (BMY1, DY011386, AT4G15210), also gradually increased their expression level at this stage.

Nine clusters do not seem to peak at any particular developmental stage and therefore do not fall into any of the above groups. Cluster 3, by far the largest cluster, contains 423 unisequences that show rapid transcript accumulation from the globular-shape embryo to the heart-shape embryo stage, but only minor changes between the heart-shape embryo and cotyledon stages. This cluster includes genes encoding enzymes involved in fatty acid biosynthesis, such as 3-ketoacyl-acyl carrier protein reductase (CN731455, AT1G24360), pyruvate dehydrogenase (CN737211, AT1G01090), acyl-(acyl-carrier protein) desaturase (DY004112, AT2G43710) and β-hydroxyacyl-ACP dehydratase (CN734884, AT5G10160). Several genes that were represented by the most abundant ESTs in our endosperm EST dataset, including those in the lipid transfer protein (LTP) family (CX266460, AT1G62790; EE435630, AT3G08770; EE541123, AT4G30880; EE543887, AT5G38160; EE541128, AT5G38195 and CN732718, AT5G64080) and the putative plastocyanin-like domain-containing proteins (CN737293, AT2G23990; CN730000, AT2G25060; CN731273, AT4G31840; CN736723, AT4G32490; CN735105, AT5G15350 and CN737110, AT5G57920), also exhibited enhanced expression levels from the globular-shape embryo to the heart-shape embryo and cotyledon stages.

#### Identification of commonly expressed genes

To identify genes that are commonly expressed across the three endosperm developmental stages, genes with expression signal values of 5000 or higher across the three stages were selected. We identified a total of 429 unisequences (see Additional file 8). In this group, 225 unisequences have similar expression levels across the three stages. The majority (>85%) of this group of unisequences have at least one identified EST. A significant number of unisequences are involved in the chloroplast, ribosome, mitochondrion and other subcellular components.

### Integrative analysis

#### Featured metabolic pathways in endosperm

Based on EST, protein profile and microarray data, we performed integrated data mining to uncover signature embolic pathways in the endosperm. The unisequences from the cDNA library and microarray and the protein entries identified by protein profile were searched against the Kyoto Encyclopedia of Genes and Genomes (KEGG) enzyme database [32] and the TAIR pathway database [33]. These searches identified 834 unigenes matching 472 enzymes (ECs) in our library. The protein profile analysis produced 161 enzymes, which corresponded to 252 unigenes. The microarray detected a total of 152 enzymes (181 unigenes) among the significantly differentially expressed unisequences (see Additional file 9). Combining all three datasets, several prominent metabolic processes in the endosperm emerged. These processes are discussed separately below.

#### Active starch metabolism status in endosperm

**Figure 5:**
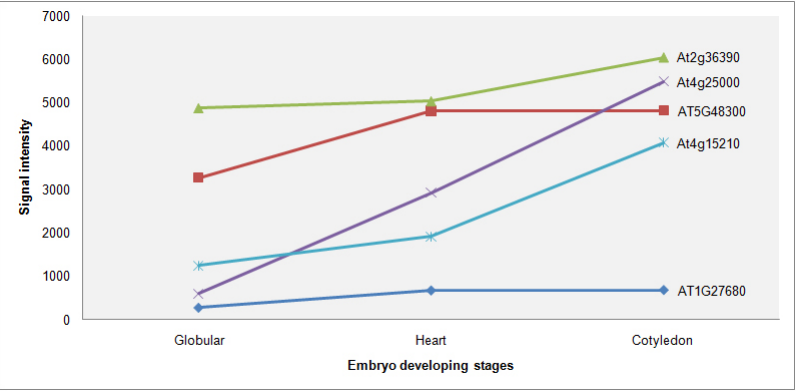
**Patterns of representative starch metabolism-related genes in endosperm**.

Both the EST dataset and the protein profile analysis revealed an abundant presence of photoassimilation machinery in the endosperm. When examined via microarray, we found a consistent trend of decreased expression of photosynthesis-related genes during endosperm development. The highest level of expression was at a phase when the endosperm accompanied the globular-shape embryo stage embryo. In keeping with the large number of starch granules present in the tissue collection, we observed genes and gene products for enzymes required for starch synthesis and starch degradation. A rate-limiting step for starch biosynthesis is mediated by ADP-glucose pyrophosphorylase (AGPase), which is composed of tetramers of small (APS) and large subunits (APL). As shown in Figure 5, a gene for APS (DY009986, AT5G48300), which encodes the regulatory subunit of AGPase, displayed increased expression at the heart-shape embryo and cotyledon developmental stages. A similar trend was also found for an AGPase large subunit gene (CN726688, AT1G27680). Likewise, another important component of the starch biosynthesis system, starch branching enzyme (CN726989, AT2G36390), displayed increased expression during endosperm development. When we looked into the expression profiles of starch degradation enzyme genes, e.g., α-amylase (CN727248, AT4G25000) and β-amylase (DY011386, AT4G15210), we also observed an increase in expression as endosperm development proceeded (Figure 5). These results suggest that there is an active metabolic flux from photoassimilation to primary carbon metabolism in the endosperm.

#### Sugar metabolism in endosperm

The channeling of sucrose and its conversion to hexose are pivotal metabolic events in the endosperm. These processes concern not only carbon supply but also osmotic regulation in the endosperm [1,34,35]. Cleavage of sucrose to hexoses can occur through two routes, mediated either by sucrose synthase (SuSy) or by invertase. Several ESTs for SuSy genes were present. SuSy isoforms were also detected in our protein profile analysis, which is consistent with a recent report on the immunolocalization of SuSy in the *Arabidopsis *endosperm [36]. The expression profile of SuSy was categorized under cluster 23 in our microarray data, where its expression at the heart-shape embryo stage was twice as high as the expression at either the preceding or later developmental stages. It was recently reported that in the liquid fraction of early seed development, hexose reaches its highest level at 10 days after anthesis (DAA) and declines afterwards [34]. It is important to note that at 10 DAA the seed also reaches its optimum size due to the presence of large vacuoles, which are sustained by accumulation of osmoticum, such as hexose. Within this context, it is tempting to speculate that the expression of SuSy in the endosperm contributes to the temporal accumulation of hexose, thereby having some relation to seed size regulation. SuSy genes are also highly expressed in the developing embryo, but their expression continues to increase after accumulation of storage products in the cotyledon [37].

In addition to SuSy, invertase activity was reported as being particularly high in the endosperm [1]. Surprisingly, however, we failed to detect invertase in either the ESTs or the protein profile datasets. Our microarray did include two unisequences corresponding to cytosolic invertases. However, the signal from those unisequences was very low in comparison to the signal for SuSy.

With regard to general carbon metabolism, it is worth noting that ESTs corresponding to the homolog of *Arabidopsis *SNF1-related kinase 3.14 (SnRK3.14), also known as CBL-Interacting protein kinase 6 (CIPK6) were frequently detected, making it the most abundant protein kinase in the endosperm EST dataset. The expression of this protein kinase gene falls into cluster 3 of the microarray data. The highest level was reached at the heart-shape embryo stage and declined at the cotyledon stage. The SnRK3s have an N-terminal catalytic domain similar to that of the yeast SNF1 kinase and also interact with calcium-binding protein calcineurin. Like SNF1 in yeast, SnRKs in crop plants have frequently been implicated in metabolic regulation in response to nutritional and stress signals [38].

#### Endosperm contains inositol phosphate and phytate metabolism

Phytate (phytic acid) is the most abundant myo-inositol phosphate (hexakisphosphate) in plant cells. It serves as a storage form of myo-inositol, providing phosphate and mineral nutrients for utilization during seed germination and seedling growth [39]. It is also one of the undesirable constituents in *Brassica *seeds, and its accumulation is known to take place in *Arabidopsis *endosperm [40]. There are at least two major routes of phytate biosynthesis: a lipid-dependent route that uses Ins(1,4,5)P_3 _produced from phosphatidylinositol 4,5-biphosphate by phospholipase C (EC3.1.4.11, 2 ESTs), and a lipid-independent route relying on the phosphorylation of myo-inositol or Ins(3)P [41,42]. In the lipid-independent pathway, Ins(3)P_1 _can be obtained from the conversion of glucose 6-phosphate, catalyzed by myo-inositol-1-phosphate synthase (MIPS, EC5.5.1.4), which is the first and rate-limiting step in the biosynthesis of all inositol-containing compounds. The EST dataset contained 35 EST members encoding MIPS. Multiple isoforms of the MIPS proteins were also found in our protein profile analysis, consistent with the detection of this enzyme in the endosperm through immunoelectron microscopy [43]. We also observed expression of other genes in the same pathway, including inositol polyphosphate multikinase (IPMK, EC2.7.1.151) and 1D-myo-inositol tetrakisphosphate 5-kinase, which phosphorylates Ins(3,4,5,6)P_4_(EC2.7.1.140). When examined via microarray, expression of the MIPS gene exhibited a drastic and continual increase from the globular-shape embryo stage to the cotyledon stage (cluster 13 of microarray data) (Figure 4). These results extends the observations of Otegui et al [40] and Mitsuhashi et al [43] and suggest that metabolic engineering efforts aimed at reducing phytate content in *Brassica *must consider endosperm metabolism and development.

#### Endosperm contains various enzymes for methyl recycling activities

Our integrated data mining also revealed robust methyl recycling metabolic activities in the endosperm. Utilization of the methyl group of S-adenosyl-methionine (SAM) is coupled by recycling of the homocysteinyl moiety and regeneration of methionine. SAM-dependent methylation events are involved in many essential metabolic processes, including chromatin modification, transcript capping, phospholipid biosynthesis and lignification of the cell wall [44]. An entire set of methylation cycle enzyme genes was previously described in barley endosperm [45]. Significantly, three major components of the methylation cycle, a cytosolic methionine synthase (EC2.1.1.14), the S-adenosyl-l-homocysteine hydrolase (SAHH2, EC3.3.1.1) and S- adenosylmethionine synthetase (EC2.5.1.6), were highly expressed based on both the number of ESTs and the microarray data. In keeping with the EST data, SAHH2 and methionine synthase were also detected in our protein profile analysis. Microarray data, on the other hand, show that the expression of these genes did not follow the same pattern. Methionine synthase gene expression was consistently at a high level and did not fluctuate during the different developmental phases. SAHH2 expression was induced at the heart-shape embryo stage and slightly increased afterwards. The expression of the methionine adenosyltransferase was highest at the heart-shape embryo stage.

It was noteworthy that ESTs corresponding to adenosine kinase 2 (ADK2, EC2.7.1.20) were also detected, although there were only three ESTs. The presence of ADK was further substantiated by its detection in the protein profile. The combined activities of ADK and SAHH2 are essential for generating lignin precursors. Within this context, it is interesting that the expression of ADK2 was in a similar pattern when compared with that of SAHH2. It has previously been reported that in the seed coat, SAHH was highly expressed, but ADK was hardly detectable [46]. Thus, assuming that metabolite trafficking occurs between the endosperm and the integument, the absence of ADK would allow speculation that the lignin precursors for the seed coat are generated from the adjacent endosperm. Earlier observation indicated that at least in castor bean, the adenosine generated through the salvage pathway by ADK is mobilized from the endosperm to the developing cotyledon [47]

#### Transcription factors (TFs) in endosperm

A search against the Database of Arabidopsis Transcription Factors (DATF) [48] identified 216 TFs in the EST collection, which can be classified into 36 families (Table 2). The details are displayed in Additional file 10. The largest group, with 73 ESTs corresponding to 11 unigenes, consists of TFs containing CCAAT-box-related motifs, to which *LEC1 *belongs. In this group, a homolog of Hap5c (AT1G08970) was detected through protein profile analysis. Other abundant transcription factor genes expressed in the endosperm include those in the AP2-EREBP family. *WRI1 *[49] is a member of this group.

**Table 2:** Transcription factors identified in the endosperm cDNA library.

TF families	No. of Loci	No. of unisequences	No. of ESTs
ABI3-VP1	7	9	11
AP2-EREBP	9	15	33
ARF	4	4	4
ARID	1	1	1
AUX-IAA	3	3	6
BBR-BPC	1	1	1
bHLH	12	20	30
bZIP	16	19	22
CCAAT-Dr1	1	1	2
CCAAT-HAP2	2	2	3
CCAAT-HAP3	5	7	61
CCAAT-HAP5	4	6	9
CPP	2	2	2
E2F-DP	2	3	3
EIL	1	3	3
GARP-ARR-B	1	2	2
GARP-G2-like	8	9	12
GRAS	3	4	4
HB	16	22	23
HMG	5	10	15
HSF	1	1	1
JUMONJI	5	5	6
LIM	1	1	1
MADS	12	16	18
MYB	13	18	20
MYB-related	6	25	30
NAC	9	15	16
other	24	32	48
PcG	2	2	2
SBP	1	1	1
TCP	2	2	2
TLP	5	8	10
Trihelix	5	7	8
WRKY	7	8	12
ZF-HD	1	1	1
zinc finger	19	25	34
Total	216	292	437

**Note: **In our endosperm cDNA library, 9,963 unique sequences (unisequences) were subjected to BLASTN against TAIR at <e^-20^, 7,585 unisequences can match *Arabidopsis *AGI identifiers. These AGIs represent 5,159 unigenes. Among these unigenes, 216 encode transcription factors. These TFs could be classified into 36 families according to the categroies of the website . The detail of each family was displayed as Additional file 10.

##### Abundance of *LEC1 *during endosperm development

**Figure 6:**
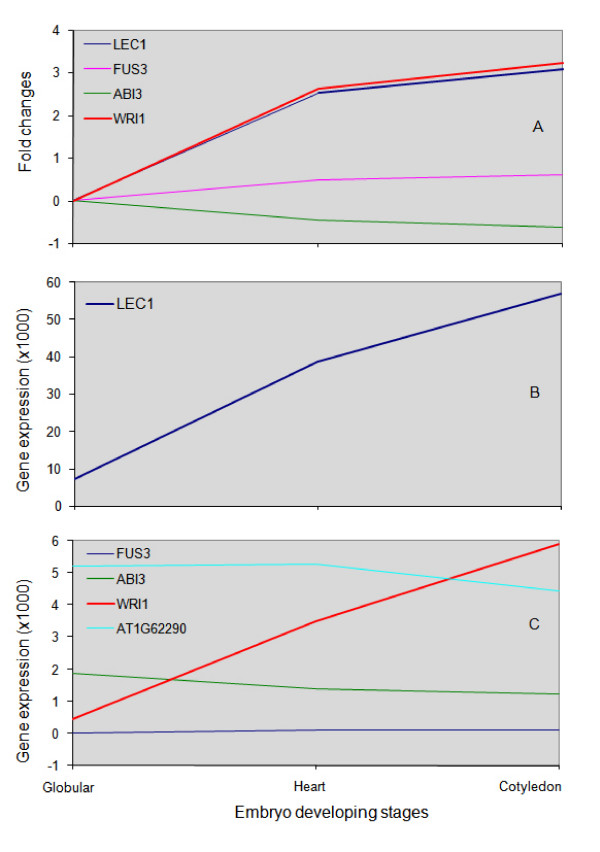
**Gene expression patterns of *LEC1 *and its targets**. (A) Uses globular as a reference point, and the subsequent fold changes (log2 ratio) are revealed in the curve. Note the one order of magnitude difference in scale of the *y *axis between (B) and (C).

**Figure 7:**
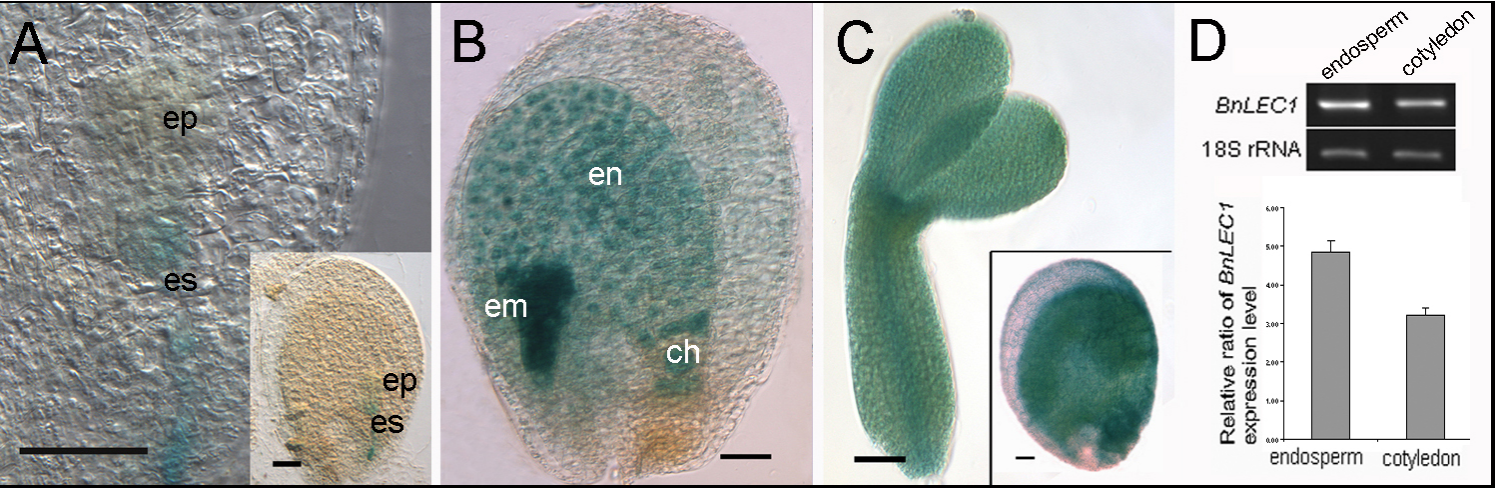
**Expression pattern of *LEC1 *in *Arabidopsis *and *B. napus***. A-C, GUS analysis of *BnLEC1 *promoter in *Arabidopsis*. (A) globular-shape embryo stage; (B) heart-shape embryo stage; (C) cotyledon stage. Insets in (A) and (C) are whole seed pictures. ep, embryo proper; es, embryo suspensor; em, embryo; en, endosperm; ch, chalazal zone. Bars = 20 μm. (D) Expression levels of *BnLEC1 *in endosperm and cotyledon tissues at the cotyledon stage of *B. napus*.

*LEC1 *was represented by 57 ESTs in our EST library and was highly expressed across the three embryo stages based on microarray data (Figure 6B). Similarly, we found another transcription factor, *WRI1*, to be highly represented by ESTs. *WRI1 *expression progressively increased during endosperm development and was highest at the cotyledon stage (Figure 6C). It has been established that WRI1 regulates the expression of several lipid biosynthesis genes. The presence of the *LEC1 *transcript in the endosperm of *Arabidopsis *was previously documented by *in situ *hybridization [50,51]. Nonetheless, the transcript could originate in the cotyledon if it is mobile between the endosperm and the embryo. We thus examined the promoter activity of *LEC1 *using a GUS reporter fusion in *Arabidopsis*. We assumed that the promoter activity of its *Arabidopsis *ortholog should serve as valid references and thus conducted experiments using the promoters of the *Arabidopsis LEC1 *in *Arabidopsis *transgenic plants. As shown in Figure 7A, *LEC1 *promoter activities were first detected in the embryo suspensor at the early globular-shape embryo stage. At the heart-shape embryo and cotyledon stages, *LEC1 *promoter activities were observed throughout the endosperm as well as the entire embryo (Figure 7B, C). This is consistent with *in situ *hybridization results reported previously [52] and in line with the results of our microarray analysis (Figure 6B). To comparatively assess the transcript levels of *LEC1 *in the embryo and endosperm of *B. napus*, we performed RT-PCR with RNA samples from endosperm and embryos at the cotyledon stage. Our results show that *LEC1 *transcript levels were higher in the endosperm than in the embryo (Figure 7D), suggesting that the expression of *LEC1 *is robust in the endosperm at this stage.

##### *LEC1 *regulatory cascade in the endosperm

Intrigued by the finding that a transcription factor required for the specification of cotyledon identity and the completion of embryo maturation is so abundant in the transcriptome of endosperm [51,53,54], we decided to look into the expression of downstream targets of *LEC1 *in the endosperm. *LEC1 *is a master transcription factor that regulates several other key transcription factors, such as *LEC2*, *FUSCA3 *(*FUS3*), *ABSCISIC ACID INSENSITIVE 3 *(*ABI3*) and *ABI5 *[55]. *LEC1 *also regulates *WRI1*, either directly or indirectly via *LEC2*. The microarray data show that expression of *LEC1 *progressively increases from the globular-shape embryo stage to the heart-shape embryo stage and then to the cotyledon stage. *WRI1 *follows the same trend (Figure 6). However, the level of mRNA from *LEC1 *is one order of magnitude higher than that of *WRI1*, which is consistent with the EST dataset. *LEC2 *is not in our microarray. *LEC1 *itself is subjected to negative regulation by *PICKLE *(*PKL*) [55], and there is a detectable but very limited level of *PKL *gene expression across the three embryo stages. *FUS3*, *ABI3 *and *ABI5 *did not have a detectable level of mRNA in our microarray data. But one of the regulatory roles of *LEC1 *via *FUS3 *is the induction of a gene (AT1G62290) encoding an aspartyl protease family protein [56]. Indeed, microarray data indicated that a substantial amount of mRNA from this gene was present across the three stages, suggesting that the *FUS3 *regulatory cascade operates in the endosperm.

**Figure 8:**
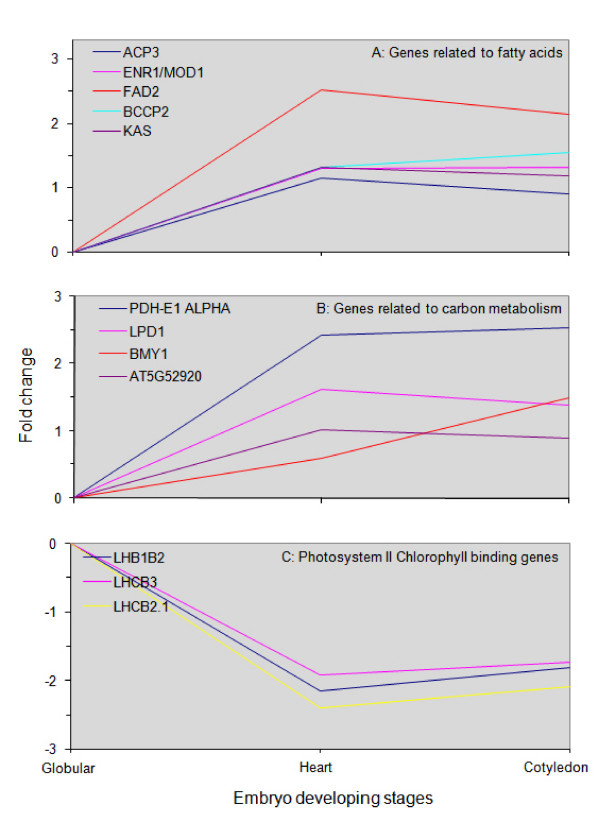
**Expression patterns of *WRI1 *target genes**. All fold changes (log2 ratio) use the globular embryo stage as a reference point.

**Figure 9:**
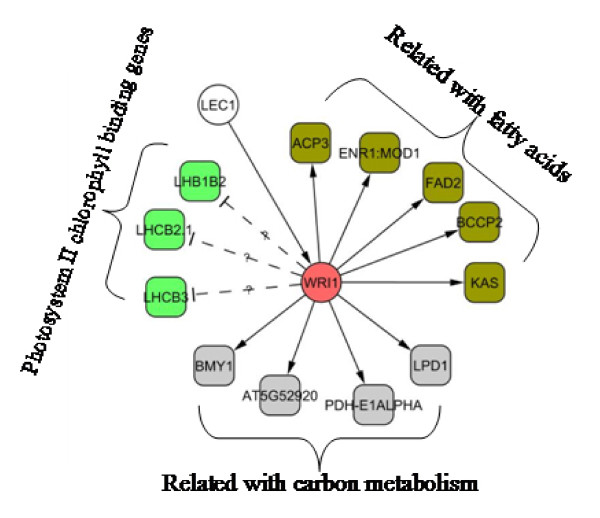
**A postulated gene regulatory network that involves *LEC1 *and *WRI1***.

The associated expression pattern of *LEC1 *and *WRI1 *provided impetus to further examine the target genes of *WRI1*. Ruuska et al. [37] investigated gene expression in the seed of the *Arabidopsis *mutant *wri1 *and found 20 genes differentially expressed between the wild type and the *wri1 *mutant. They categorized the genes into four groups: genes associated with fatty acid metabolism, genes associated with carbon metabolism, miscellaneous genes and genes up-regulated in the mutant. Our microarray data show that the expression profiles of their orthologs in *Brassica *endosperm exhibit a pattern similar to what would be expected of a *WRI1 *regulatory network, except for the photosynthesis-related genes (Figure 8). Ruuska et al. [37] reported that photosystem II chlorophyll-binding genes were down-regulated in the *Arabidopsis wri *mutant, suggesting that *WRI1 *was a positive regulator of these genes. During endosperm development, however, photosynthesis-related gene expression exhibited a downward trend after the globular-shape embryo stage, and thus there was a negative correlation with the *WRI1 *transcript level(Figure 8). Based on the information gained from this study and existing literature [37], we envision a small regulatory network in which both *LEC1 *and *WRI1 *are involved in the endosperm (Figure 9). The presence of such a regulatory network is at least consistent with regard to the synthesis of storage lipids, which were shown to accumulate to a significant amount in the endosperm of *Brassica napus *and *Arabidopsis *[3,57]. This merits further study.

## Conclusion

We reported the first comprehensive survey of the endosperm gene expression profile in a major oilseed crop. The datasets presented in this work portray a gene expression landscape that is complicated in structure but coordinated in regulation. We also provide insight into several prominent metabolic pathways in the endosperm that have implications for improving the productivity and quality of canola. Our findings on the endosperm expression of *LEC1 *and other transcription factors involved in regulating storage product biosynthesis in the cotyledon provide impetus for further investigation into the role of endosperm in controlling embryo development and storage product deposition.

## Methods

### Preparation of the cDNA library

#### Plant materials

*B. napus *DH12075 was grown in soil-based compost under standard greenhouse conditions as previously described and used for endosperm collection [58].

#### RNA extraction

Total RNA was extracted from frozen tissues at each embryo developmental stage using RNeasy Plant Mini Kit (Qiagen, Mississauga, ON, Canada) and on-column DNase digestion with RNase-free DNase Set (Qiagen) was performed for each sample during RNA isolation according to the protocol provided by the manufacturer. mRNA was isolated using the Dynabeads mRNA Purification Kit (Invitrogen). The yield and RNA purity were determined using a Nanodrop ND-1000 Spectrophotometer, and the intactness of RNA was verified by Agilent 2100 Bioanalyzer. Either the total RNA or mRNA was used for subsequent cDNA synthesis.

### EST analysis

#### cDNA library construction and sequencing

Two cDNA libraries were constructed using a Creator SMART cDNA Library Construction Kit from Clontech (BD Biosciences Clontech, Palo Alto, CA, USA) according to the manufacturer's recommendations. One library originated from RNA isolated from endosperms at heart-shape-embryo stage (Library II), and another one derived from RNA isolated from mixed endosperms at various developmental stages (Library I). First-strand cDNA of Library I were synthesized starting with 0.6 μg mRNA, and amplified by LD-PCR (Long Distance PCR) with 13 cycles. Similarly, first-strand cDNA synthesis for library II was started with 1.0 μg total RNA and then amplified by LD-PCR with 21 cycles. The amplified cDNAs were digested with SfiI and directionally ligated into Clontech's pDNR-LIB vector. The resulting plasmids were transformed into DH10B T_1 _Phage Resistant Cells (Invitrogen) by electroporation. The average insert size of cDNAs was determined by PCR amplification with M13F/M13R primers [59] and agarose gel eletrophoresis.

Singel bacterial colonies of the *Brassica *cDNA libraries were inoculated in 96-well microtiter plates containing 150 μl aliquots of LB medium plus 10% (v/v) glycerol and 34 μg/ml chloramphenicol. After 20–22 h incubation at 37°C, cells were either used immediately for the next step or stored at -80°C. DNA sequencing templates were prepared from 1 μl of the bacterial cell culture using the TempliPhi DNA Sequencing Template Amplification Kit (GE Healthcare) according to the protocol provided by the manufacturer. One microliter of the amplified products was used directly in a 10 μl cycle sequencing reaction. Sequencing was performed on an ABI 3730 DNA sequencer using BigDye V3.1 (Applied Biosystems) and M13F primer were used for sequencing from the 5' end of cDNAs.

#### EST assembly and gene identification

Raw sequences of *Brassica *endosperm were stored in the Fiesta database [16]. Lucy [60] and Trimpoly [61] were used to remove low-quality regions (quality score <20) as well as to trim poly-A signals. Clustering was performed using TGICL [62] with stringent parameters and in-house Perl scripts.

For functional annotation of ESTs and functional categorization of unisequences, BLASTX against the *Arabidopsis *Information Resource (TAIR, V7) protein database was run locally. The BLASTX outputs and the *Arabidopsis *functional annotations were parsed with scripts to extract information and to assign the descriptions, GO terms, and Kyoto Encyclopedia of Genes and Genomes (KEGG) enzyme classification number (EC number) of the *Arabidopsis *top-scoring hits to the corresponding *Brassica *sequences.

All information generated in the pipeline was automatically uploaded to the database of the project by other in-house scripts. This database is a searchable database [63] containing data about the project (members, groups, plant material, libraries, etc.) and all relevant information about every EST (raw and trimmed sequence, contig consensus sequences, functional annotations, orthologs in *Arabidopsis *and other organisms, BLAST results, etc.). The database can be accessed by the research community to retrieve data in a customized way. The site also provides the opportunity to search the *Brassica *EST sequences using BLAST.

### Protein profile

About 250 μl *B. napus *endosperm at the heart-shape embryo stage was prepared. The tissue was mixed with 750 μl cold acetone, grounded using pellet Pestles^® ^(Kimble-Kontes), kept at -20°C for 3 hr and centrifuged at 20,000 g at 4°C for 30 min. The pellet was air dried at 4°C overnight. The dry weight was determined at approximately 5 mg. The dried pellet was extracted with 500 μl extraction buffer (50 mM Tris-HCl, pH 8.8, 6 M urea, 2% SDS, and 5 mM DTT) at room temperature for 1 hr with stirring. After the addition of iodoacetamide to a final concentration of 10 mM, extraction was allowed to continue for another hour before centrifugation at 20,000 g for 30 min to remove tissue debris.

The extracted proteins were separated on 10~20% linear gradient Criterion Tris-HCl gels (Bio-Rad) at a constant voltage of 150 V. After visualization with Bio-Safe Coomassie Blue (Bio-Rad), the gel was excised into 30 bands. The individual gel bands were further diced and placed into a 96-well microtiter plate. The proteins were automatically de-stained, reduced with dithiothreitol, alkylated with iodoacetamide, and digested with porcine trypsin (Promega) using a MassPREP protein digest station (Micromass). The digested samples were then ready for mass spectrometry.

The tryptic peptide was analyzed on a Q-ToF Ultima Global hybrid tandem mass spectrometer (Waters) with an online CapLC ternary nanoHPLC system using a 2-hr separation program. LC-MS/MS data were processed using MASCOT Distiller software (Matrix Science) and searched against the TAIR database [64] using MASCOT Daemon (Matrix Science). Peptide matches were considered unambiguous if ion scores were significant (p < 0.05), and protein identifications included at least one top-ranking peptide match with a score above the significance threshold.

### Microarray analysis

#### *Brassica *10K cDNA array

A set of 10,642 cDNA amplicons was produced and printed in duplicate side-by-side columns on Corning UltraGAPS slides with a Chipwriter Pro microarray printer (Bio-Rad) at the Biotechnology Research Institute (BRI), National Research Council Canada (NRC). The microarray consists of 12 row × 4 column sub-microarrays, and each sub-microarray has 23 row × 22 column (506) spots. The quality and reproducibility of the microarray was tested [28].

#### RNA labeling, microarray hybridization and washing

Approximately 1 μg total RNA was treated with DNase I and reverse transcribed into aRNA with Message™ II aRNA Amplification Kit (Ambion, Austin, TX, USA) according to the manufacturer's protocol. The aRNA was selected as the template for fluorescent target preparation for microarray experiments. The quality of amplification was identified using agarose gel electrophoresis, and the aRNA yield was quantified using a spectrophotometer DU^®^7400 (Beckman, USA).

An aliquot of 3 μg of aRNA was reverse transcribed using an oligo(dT)_20 _primer and 400 units of Superscript III reverse transcriptase (Invitrogen, Cat. No.18080-044) in the presence of 25 mM dATP, 25 mM dCTP, 25 mM dGTP, 15 mM aminoallyl-dUTP (Ambion, Cat. No. AM8439) and 10 mM dTTP. The single stranded cDNA was purified using YM30 filter column (Millipore, Billerica, MA) and labeled with CyDye post-labeling dye (Amersham, UK) for 1 hr. After a second purification as above, both Cy5- and Cy3-labeled cDNAs were pooled. The mixture was concentrated using a vacuum dryer and resuspended in 30 μl hybridization buffer. The pre-hybridization and hybridization procedures were performed as previously described [65].

Two biological duplicates were set up, including four technological repeats in each duplicate. A dye-swap hybridization experiment was performed for each pair of target RNA comparisons. We performed four independent aRNA labelings for each tissue pair. For example, we used globular-shape embryo stage endosperm RNA coupled with Cy5 dye vs. heart-shape embryo stage endosperm RNA coupled with Cy3 dye, and the dye-swap experiment was with globular-shape embryo stage endosperm RNA with Cy3 dye vs. the heart-shape embryo stage endosperm RNA with Cy5 dye. Each tissue pair experiment was done with four repeats, using a total of 24 microarrays for the whole experiment.

#### Data collection and normalization

Hybridized arrays were scanned using a Genepix 4000B microarray scanner (Axon Instruments, CA, USA) at 5 μm resolution, 100% laser power, and different PMT values to obtain a similar green and red overall intensity. Raw spot fluorescence intensities were collected using GenePix Pro version 6.0 (Axon Instruments, CA, USA). A quality control filter was used to flag questionable spots on the array so they could be removed from analysis.

Before normalization, a basic pre-processing was performed. The outlier spots with median intensity larger than that of (median ± 10 × SD) were removed. Channel intensity was considered low if its background-corrected intensity value was less than a preset low-intensity threshold (LIT) of 0.5; low channel intensities were replaced by the LIT. The spot was marked as bad if either of the two channels was low. Spots with background-subtracted median intensity greater than median background intensity in at least one channel were selected and used for normalization and further analysis. Array features annotated as "DMSO," "Blank" or "Empty" were flagged and excluded from analysis. A spot was removed if the fold change of the Cy3/Cy5 group was in the opposite direction of that of the Cy5/Cy3 group. For example, if spot A was up-regulated for the Cy3/Cy5 group and down-regulated for the Cy5/Cy3 group, then spot A was removed because of its conflicting data.

#### Data analysis

Foreground intensities of selected genes were background corrected using the normexp background measure, and the signal intensities were normalized using 'global loess' for within-array normalization and quantile for between-array normalization using R-package Limma [66]. The signal intensity (background-corrected) of each spot on the microarray was combined by averaging two biological duplicates using Excel 2003 (Microsoft).

After the normalization process, the signal intensities and log2 ratio of the two channels of each replicate were obtained. These two sets of data were processed through Rank Product implemented in the BioMiner software suite [67] with a 5% false discovery rate [30,31]. Genes with very inconsistent signal intensities and log2 ratio were excluded from this process. Then we combined the results from all three stages. The genes that ranked high in both datasets were considered differentially expressed genes and classified into patterns based on the clustering approach [31]. The details are listed in Additional file 11.

#### Annotation and GO functional categorization

The DNA sequence for each of the unisequences spotted on the array was searched for in the TAIR protein database (version 7) using BLASTX programs at an E value less than 10^-5 ^[68]. The best match was extracted using an in-house Perl script and used as a basis for obtaining annotations for each probe based on sequence identity. The best matches were compared to terms of the Gene Ontology (GO) Consortium [15]. Categories were assigned based on biological, functional, and molecular annotations available from GO.

#### RT-PCR

To confirm the expression patterns obtained from microarray data analysis, semi-quantitative RT-PCR analysis was performed. The first-strand cDNA was synthesized with the SuperScript™ synthesis system (Invitrogen) and used as a template for amplification. A total of 12 cDNA probes that exhibited differential expression at least in one tissue-pair comparison were chosen for RT-PCR. An 18S rRNA coding gene was chosen as an endogenous reference (see Additional file 12). About 2 μg aRNA, which were the same as that for microarray target labeling, was reverse transcribed into first-strand cDNAs using oligo (dT)_20 _primer in a total volume of 20 μl.

Amplified PCR products (10 μl) were resolved on a 1.2% (w/v) agarose gel with 1× TBE running buffer. The images were produced and the signal intensity of each band was quantified with ImageJ software [69]. All PCR experiments were repeated independently three times. The signal intensity of each tissue band was normalized against that of the 18S rRNA transcript; then the ratio of each tissue pair was calculated, and the correlation coefficient between microarray and RT-PCR was obtained based on signal ratio.

#### Promoter::GUS construction and histochemical analysis of GUS expression

The putative promoter sequence of *LEC1 *was amplified with PCR (*LEC1*: 5'-AAGCTTTATGGGCTGCTTGTTC-3', 5'-ACTAGTGTTTCTCTGCCGTCTTTT-3') and inserted in pER330, a GATEWAY destination vector [70], in place of the 35S promoter using different restriction sites. The obtained pER330 constructs were recombined with the entry vector pER367 containing GUS protein to generate the final expression constructs in *Arabidopsis*. GUS staining of siliques was performed using the substrate 5-bromo-4-chloro-3-indolyl β-D-glucuronide as previously described [71]. Samples were incubated in a β-D-glucuronide solution at 37°C for 20 hr. After incubation, samples were cleared in a clearing solution of chloral hydrate/glycerol/water (8:1:2, w/v/v) and photographed.

## Authors' contributions

YH performed microarray hybridization, analyzed part of microarray and cDNA library datasets. LC planted materials and collected endosperm samples. LPW performed RT-PCR and promoter::reporter fusion analysis. KV assembled cDNA sequences, conducted the BLAST analysis and set up the EST database. SP, ZYL and YLP carried out part of data processing. LLW and AR conducted protein profile analysis. DQX and RD contributed to microarray analysis. JTZ designed the study and JTZ, YH and YLP wrote the manuscript.

## Supplementary Material

Additional file 1 **A list of all unisequences identified and their best BLASTX match annotations**. 9963 unisequences from endosperm cDNA library with best BLASTX match annotations, unisequence length, contig size, code in dbEST and locus ID. Click here for file

Additional file 2 **Highly abundant unisequences in *Brassica napus *endosperm**. A list of contigs derived from 10 or more ESTs. Click here for file

Additional file 3 **A table of proteins identified in endosperm via protein profiling**. A total of 809 genes identified from endosperm protein dataset. Click here for file

Additional file 4 **A table of 1229 significantly differentially expressed unisequences during endosperm development**. This table shows annotations and expression level changes of 1229 significantly differentially expressed unisequences during endosperm development. Click here for file

Additional file 5 **A table listing 24 patterns of significantly differentially expressed unisequences during endosperm development**. The data show the significantly differentially expressed unisequences list and gene expression level changes in each cluster. Click here for file

Additional file 6 **Biological functional annotation of differentially expressed unisequences in each cluster**. The data show the distribution of GO annotation in biological processes for each cluster. Click here for file

Additional file 7 **Tables listing stage-favored genes during endosperm development**. These data show stage-favored genes with expression level changes, EST accession numbers in dbEST of NCBI and annotations. Click here for file

Additional file 8 **A table listing universally expressed genes during endosperm development**. The table shows universally expressed genes with expression level changes, EST accession numbers in dbEST of NCBI and annotations. Click here for file

Additional file 9 **A table of enzyme-encoding genes identified in the endosperm**. Enzyme-encoding genes indentified in *Brassica *endosperm using cDNA library, protein profiling and cDNA microarray. Click here for file

Additional file 10 **A table of transcription factors identified in the endosperm cDNA library**. Unisequences encoding transcription factors in the endosperm identified from cDNA library. Click here for file

Additional file 11 **A file describing methods for microarray data analysis**. Details for microarray data analysis Click here for file

Additional file 12 **A table listing primers for RT-PCR validation of the selected unisequences**. Primers for RT-PCR validation of the representative unisequences identified by cDNA microarray. Click here for file
