# Supplementary material for: Probing the endosperm gene expression landscape in Brassica napus
Source: BMC Genomics. 2009 Jun 2;10:256. doi: 10.1186/1471-2164-10-256 (PMC2702316; doi:10.1186/1471-2164-10-256)
Supplement: Additional file 11 — A file describing methods for microarray data analysis. Details for microarray data analysis [file 1471-2164-10-256-S11.docx]

### Additional file 11: Methods for microarray data analysis

1. **Data pre-processing**

After the normalization process, the (background-corrected) signal intensities and log2 ratios of the two channels of each replicates are obtained. For each of the three stages (G=globular, H=heart, C=cotyledon), there are 16 replicates for signal intensities and 8 for the log2 ratios. These two sets of data are processed through rank product implemented in the BioMiner software suite with a false discovery rate (FDR) < 0.05 [1, 2].

This resulted in 3047 genes from the signal intensity, and 2503 genes from log ratio data. They are considered significant in each set [2]. By joining (overlap of) these two sets of so called significant genes, we identified 1229 genes which are ranked high in both datasets. We call these 1229 genes as significantly and differentially expressed genes, and classify them through pattern based clustering approach [3].

The beauty of rank product is to remove those genes with significant inconsistent ranking either in the log ratio or in the signal intensity. The select genes are consistent in ranking across various replicates of either log ratios or signal intensities of the same probes. However the consistent ranking does not guarantee consistent log ratio or signal intensity. We did notice a few inconsistent spots. Thus we did further filtering as below.

We first remove the few spots (<2%) that are flagged as “questionable” in the array scanning process. Since each probe is double sported on the array, we combined the duplicate spots and performed further filtering. This combination resulted in 16 replicates for the log ratio data and 32 replicates for the signal intensity data. Most genes with very inconsistent values among the replicates are removed as below.

For the log2 ratio values, a spot is removed if fold-change of the Cy3/Cy5 group is in the opposite direction of that of Cy5/Cy3 group. For example, if the Cy3/CY5 group is up-modulated (mean log2 ratio >= 0.5) but the Cy5/Cy3 group down-modulated (mean log2 ratio <= -0.5), this probe is removed. Through this process, 110 log ratios are removed and labelled as “missing”, out of these, 86 are attributed to C/H ratio. Nevertheless, the final number of genes remains to be 1229 for the subsequent data analysis.

For the signal intensity analysis, we first find median among the replicates. Then we take the log2 ratio of each replicate over the median. A replicate is considered as outlier if its log2 ratio value is larger than or equal to 2 (4 times as high as the median) on one hand, or smaller than or equal to -2 (a quarter of the median) on the other. After this further filtering process, the means and medians generally became very close even a few small perceivable differences exist. These small differences are usually attributed to dye swapping and we have to live with it in the following analyses. To avoid potential bias toward dyes, we calculated the mean rather than using median of the remaining replicates. If a probe had less than half replicates remaining or if in the remaining replicate, the mean and median are marked different [abs(log2 ratio)>=2], we remove that value and label that mean as missing value. After this process, less than 0.1% (9) is labelled as “missing”.

1. **Data analysis**

We first perform pattern based clustering [3]. We defined two thresholds for this classification process, 0.5 for low change cut off and 1.0 for changes. Two time intervals can be considered for this experiment, from globular-shaped-embryo to heart-shaped-embryo stages and from heart-shaped-embryo to cotyledonary stages. There were five categories of changes in each time interval (Table 1)

We defined fold change from globular-shaped-embryo to heart-shaped-embryo stages by the log2 ratio of H/G, and from heart-shaped-embryo to cotyledonary stages by the difference between two log2 ratios:

Log(C/H) = Log(C/G) – Log(H/G) (1)

We did not use C/H directly because substantial number of data points in that experiment was removed due to inconsistence between the dye swaps and noisiness among the replicates (data preprocessing section). Among the two time intervals, 25 patterns of changes can be identified (Table 2).

1. **Identification of stage-specific genes**

From the clusters detailed in Table 2, we are able to identified three groups of stage favoured gene clusters (Table 3).

In order identify the stage specific genes, we looked into the gene expression signals of these group of clusters in addition to the log2 ratio values. To bring the gene expression signals to a comparable scale, we divided gene expression signals value by 1000. The signal level of below 1000 can be considered very little expression, a value of 5000 and higher is considered significant expression and a value of 10,000 or higher is considered highly expressed. An expression level between 1000 and 5000 is considered moderate.

This combination of the log2 ratio data with the gene expression signals allows us to further classify the three groups in order to identify the stage specific genes (Table 3). A stage-specific gene is defined as that the gene is significantly expressed in the stage, but very moderate, if any, expression in the other two stages. We used divisive hierarchical clustering with coefficient of divergence (Equation 2) as a distance measure.

$d_{ik}=\sqrt{\left(\sum_{j\in A} \left(\left(\mathbf{x}_{ij}-\mathbf{x}_{kj}\right)/\left(\mathbf{x}_{ij}+\mathbf{x}_{kj}\right)\right)^{2}\right)*\left(p/c\right)}$ (2)

- $A=\{j|j\in\{1..n\}\wedge\text{attribute value }\mathbf{x}_{ij}\text{ is} \text{not} \text{missing}\}$,
- *p* = 1 or n, c = [1..n];
- where both X*_ij_* and X*_kj_* are not missing value, ‘c’ is the number of variables for which neither X*_ij_* nor X*_kj_* is missing and ‘n’ is the total number of variables for certain attribute. ‘p’ is the denormalized coefficient that could be 1 or n.

We have investigated many other distance measures, such as Euclidean and Difference in Shape for distance measure. We also tried unsuccessfully using k-mean clustering coupled with stability and general stability as a guideline for k value. None of them worked as well as what is finally used [4].

Table 1. Categories of fold change (log2 ratio) in each time interval

| **Change** | **Range** |
| --- | --- |
| No-chang | AND(>-0.5, <0.5) |
| lo-rise | AND(>=0.5, <1.0) |
| rise | > = 1.0 |
| fall | < = -1.0 |
| lo-fall | AND(>-1.0, <= -0.5) |

Table 2. Patterns and the number of genes identified in the pattern based clustering process

| Cluster ID | Interval 1 (H/G) | Interval 2 (C/H) | Number of genes |
| --- | --- | --- | --- |
| 1 | No-chang | No-chang | 10 |
| 2 | lo-rise | No-chang | 18 |
| 3 | rise | No-chang | 423 |
| 4 | fall | No-chang | 351 |
| 5 | lo-fall | No-chang | 21 |
| 6 | No-chang | lo-rise | 16 |
| 7 | lo-rise | lo-rise | 13 |
| 8 | rise | lo-rise | 42 |
| 9 | fall | lo-rise | 65 |
| 10 | lo-fall | lo-rise | 7 |
| 11 | No-chang | rise | 7 |
| 12 | lo-rise | rise | 1 |
| 13 | rise | rise | 7 |
| 14 | fall | rise | 23 |
| 15 | lo-fall | rise | 2 |
| 16 | No-chang | fall | 4 |
| 17 | lo-rise | fall | 10 |
| 18 | rise | fall | 71 |
| 19 | fall | fall | 2 |
| 20 | lo-fall | fall | 0 |
| 21 | No-chang | lo-fall | 13 |
| 22 | lo-rise | lo-fall | 16 |
| 23 | rise | lo-fall | 97 |
| 24 | fall | lo-fall | 7 |
| 25 | lo-fall | lo-fall | 3 |

Table 3. Group of stage favored clusters

| Embryonic stage | Clusters |
| --- | --- |
| Globular-shape embryo | 4, 5, 9, 19, 24, 25 |
| Heart-shape embryo | 17, 18, 22,23 |
| Cotyledon | 7, 8, 11, 12, 13 |

**References:**

1. Pan Y, Pylatuik JD, Ouyang J, Famili AF, Fobert PR: **Discovery of functional genes for systemic acquired resistance in Arabidopsis thaliana through integrated data mining**. *Journal of bioinformatics and computational biology* 2004, **2**(4):639-655.

2. Breitling R, Armengaud P, Amtmann A, Herzyk P: **Rank products: a simple, yet powerful, new method to detect differentially regulated genes in replicated microarray experiments**. *FEBS letters* 2004, **573**(1-3):83-92.

3. Phan S, Famili F, Tang Z, Pan Y, Liu Z, Ouyang J, Lenferink A, O'connor MM-C: **A novel pattern based clustering methodology for time-series microarray data**. *International Journal of Computer Mathematics* 2007, **84**(5):585-597.

4. Famili AF, Liu G, Liu Z: **Evaluation and optimization of clustering in gene expression data analysis**. *Bioinformatics* 2004, **20**(10):1535-1545.
