# Supplementary material for: Probing the endosperm gene expression landscape in Brassica napus
Source: BMC Genomics. 2009 Jun 2;10:256. doi: 10.1186/1471-2164-10-256 (PMC2702316; doi:10.1186/1471-2164-10-256)
Supplement: Additional file 12 — A table listing primers for RT-PCR validation of the selected unisequences. Primers for RT-PCR validation of the representative unisequences identified by cDNA microarray. [file 1471-2164-10-256-S12.docx]

**Additional file 12. Primers of the selected unisequences for validation with RT-PCR**

| Genes | Forward primers (5’-3’) | Reverse primers (5’-3’) |
| --- | --- | --- |
| 8RDBRH_UP_009_D12_12SEP2003_090 | CATTACCAATATCCTTACGCA | TATAACGCCACATTTGGCTAC |
| 24RDBNH_UP_029_B02_12JAN2004_014 | TGGTATGATCTTCGTCCTCGTC | CTCAAAATGTACTGATGGTAAGC |
| 26RDBNT_UP_023_D05_22JAN2004_041 | TGTCTTTGTTGTGTGAAGGCA | ACCAAAAGGACTAATGGAAGA |
| 25RDBNM_UP_003_C01_14JAN2004_011 | TTCCTTCCCTACCTTGACACCTT | TACGAAGGACATAGCACCGAGT |
| 26RDBNT_UP_014_F02_21JAN2004_006 | TGAAGCACCGACAGTGACAATA | CCCAATAAACGCTCTTTTGATC |
| 6ETGS6H_UP_001_C04_15MAY2003_014 | ATTCTCTTCACTTCTCTCTGTCA | GAAATCATGCTTGAATGCTTCC |
| 26RDBNT_UP_018_H05_21JAN2004_033 | GAGTTTGGGGTTTGCAATTGGG | CTGATGGCTTGGAGACAGAGTC |
| 26RDBNT_UP_010_E10_20JAN2004_072 | AGCACTGTAATGACTACACTGCC | AATAGCACCAATGTTTTTGAGAC |
| 9RDBNGA_UP_003_F04_26SEP2003_022 | AAAGGCTTCCATTTACTTCGC | CATCCAACAAGATGATGATACTG |
| BNDH5DCT_UP_026_D08_28JAN2004_058 | AAACCACAAGACACACCGCAGA | ATCACAGCTCCAACCAGCGTAT |
| BNQA_UP_020_A08_22SEP2003_064 | TTGAGAAGAAAGGTTTGACTGC | GGCTGGGTTTGTCTTACAGAA |
| 24RDBNH_UP_007_G10_19DEC2003_068 | GTTCTTTCAGATTGATGGTTACT | GTTTGATGAGAGAGGACATATTAC |
| LEC1 | AGCTCCTCTCTCTCACTATCAG | CCAAAGGATATCTTCAGCAGTT |
| 18S rRNA | GGTAGGCGATTGGCTAAATTGTCTGC | GAGACACCAACAGTCTTTCCTCTGCG |
